# Exploring pembrolizumab-induced hidradenitis suppurativa: A case-report

**DOI:** 10.1016/j.jdcr.2025.02.035

**Published:** 2025-03-14

**Authors:** Andrea B. Lastra-Annexy, Mario G. Lozano-Franco, Alma M. Cruz-Santana

**Affiliations:** a Universidad Central del Caribe, Bayamón, Puerto Rico; b Department of Dermatology, University of Puerto Rico Medical Sciences Campus, San Juan, Puerto Rico

**Keywords:** hidradenitis suppurativa, immune checkpoint inhibitors, pembrolizumab

## Introduction

Pembrolizumab (Keytruda), an immune checkpoint inhibitor (ICI), is a humanized monoclonal anti-Programmed cell death protein (PD-1) antibody that is used in the treatment of many malignancies. PD-1, expressed on immune cells like T cells, B cells, and natural killer cells, regulates the immune response by inhibiting it when bound to its ligands, PD-L1 or PD-L2. This mechanism helps maintain immune tolerance and prevent autoimmunity. However, cancer cells and pathogens can exploit the PD-1/PD-L1 or PD-1/PD-L2 pathways to evade immune detection.^1^ ICIs such as pembrolizumab block this interaction, restoring T cell activity against cancer and simultaneously, stimulating cytokine production (eg, IL-2, IL-6, IL-17, IFN-gamma, and TNF-alpha).^2^ This enhanced immune response can lead to immune-related adverse events, with cutaneous reactions being the most common. The most common immune-related adverse events reported include vitiligo, pruritus, morbilliform, eczematous, lichenoid, and psoriasiform eruptions; most are mild and have resolved with the discontinuation of the immunotherapy.^3^ Here, we present a patient who developed hidradenitis suppurativa (HS) during anti-PD-1 (pembrolizumab) therapy.

## Case presentation

**Fig 1:**
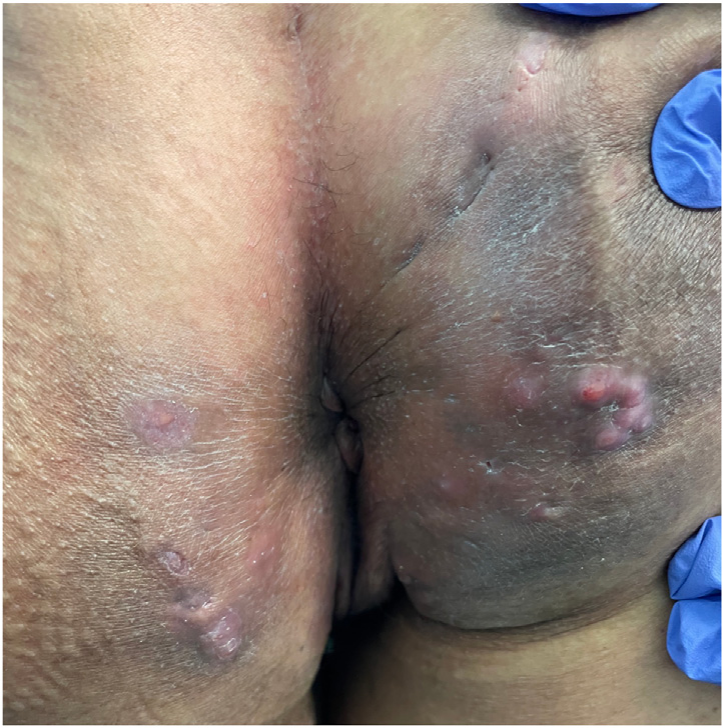
Interconnected draining fistula on the left gluteal cleft and 3 interconnected draining fistulas on the right gluteal cleft.

**Fig 2:**
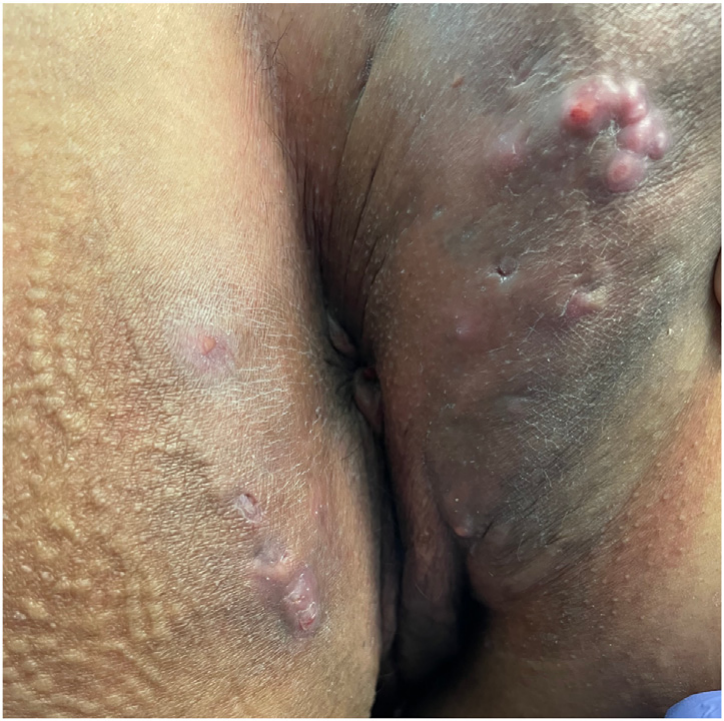
Interconnected draining fistula on the left gluteal cleft and 3 interconnected draining fistulas on the right gluteal cleft.

**Fig 3:**
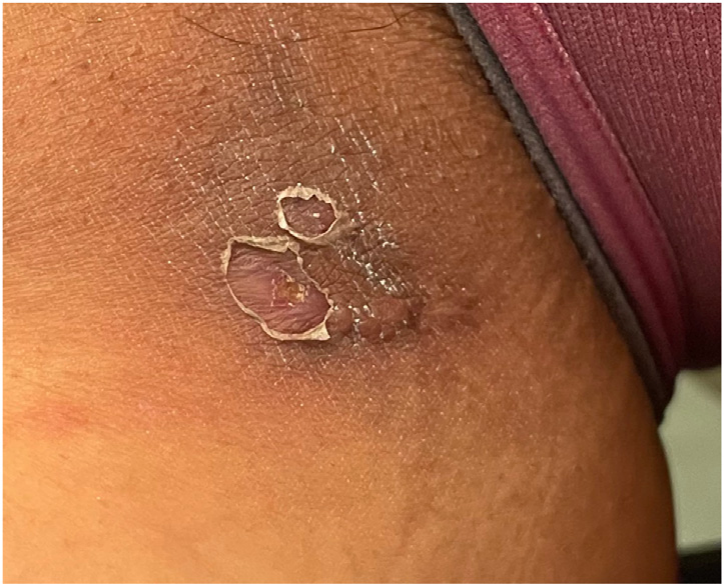
Noninflammatory nodule on the right proximal thigh.

A 48-year-old nonsmoking female with a BMI of 23.8 kg/m^2^ and stage IV cervical cancer was treated with pembrolizumab. She received 100 mg (or 2 mg/kg) of pembrolizumab every 3 weeks for 24 months, totaling 32 cycles. Three months after initiating the infusions, she developed recurrent painful nodules and abscesses in her right gluteal cleft. Soon after, similar cutaneous lesions appeared on her left gluteal cleft and right proximal thigh. Physical examination revealed one interconnected draining fistula on the left gluteal cleft, 3 interconnected draining fistulas on the right gluteal cleft (Figs 1 and 2) and one noninflammatory nodule on the right proximal thigh (Fig 3).

The patient reported that the lesions were intermittent, flaring up acutely 1 week after each pembrolizumab infusion and resolving by the time of the next infusion. During active flare-ups, she experienced pain rated at 8 of 10, suppuration at 5 of 10, and pruritus at 8 of 10. She denied having similar cutaneous lesions prior to pembrolizumab therapy and had no personal or family history of HS or other risk factors. Skin culture from one of the draining fistulas revealed mixed flora, including Escherichia coli and Campylobacter spp. Physical examination was also notable for a depigmented patch, consistent with a diagnosis of vitiligo, which she reported developing 1 month after starting pembrolizumab therapy.

A suspected diagnosis of pembrolizumab-induced HS, stage Hurley 2, was made. The patient was prescribed minocycline 100 mg daily for 6 weeks, along with Diclofenac Gel 3%, Clindamycin Gel 1%, Chlorhexidine gluconate 4% skin cleanser, zinc, and turmeric. Two months after completing her final cycle of pembrolizumab, the patient has not experienced any reactivation of lesions, with only postinflammatory hyperpigmentation and scarring remaining.

## Discussion

HS is a chronic inflammatory skin condition characterized by lesions that include nodules (inflammatory or noninflammatory), abscesses, fistulas (draining or nondraining), and/or scars (rope-like or fibrosing). These lesions are predominant in intertriginous areas rich in apocrine glands such as the axilla, inframammary, groin, perianal, and perineal. This condition is associated with smoking, obesity, metabolic syndrome, increased androgens, and the risk is higher in individuals with a first-degree family relative with HS.^4^ The main defect in HS development is the occlusion and inflammation of a hair follicle, where the mechanism behind follicular occlusion is still being investigated. Recent research by Melnik and Plewig propose that HS is an auto-inflammatory disease linked to the gamma-secretase/Notch pathway dysregulation; a pathway crucial for maintaining hair follicles and skin structures. Disruption of this pathway can lead to the formation of cysts, disrupt gland balance, and trigger chronic inflammation through innate immune responses, including elevated proinflammatory cytokines such as TNF-alpha, IL-1, and IL-17. In HS, altered toll-like receptor signaling in immune cells such as macrophages and dendritic cells increase cytokine production. Activated dendritic cells lead to IL-23 secretion which promotes T helper-17 (Th-17) cell polarization and thus, the release of more pro-inflammatory cytokines such as IL-17. Chronic HS lesions have been found to show the infiltration of IL-17-producing Th-17 cells in the dermis.^5^

Due to its nonspecific immune activation, the use of ICIs may contribute to the development of immune related adverse effects, especially cutaneous immune adverse effects. ICIs therapy have reported the development or exacerbation of neutrophilic skin diseases, including pre-existing psoriasis and primarily neutrophilic conditions such as acute generalized exanthematous pustulosis and Sweet syndrome.^6^ Anti-PD-1 therapy releases immune system inhibition, which can control tumor progression but can also trigger T cell-mediated adverse events, possibly Th-17 mediated. This can result in the recruitment of neutrophils in tissues. Considering that IL-17 plays a crucial role in HS and that Th-17 cells are elevated in HS-affected skin, there are indications that an IL-17 axis might be associated with adverse effects caused by ICIs.^7^ If so, HS should be considered as a possible cutaneous immune adverse effect triggered by the use of ICIs, as seen in this case report. Reports of induced HS are rare, with only one other case thus far described.^7^ To our knowledge, this is first description of pembrolizumab associated with HS. Clinicians should be aware of this potential association and further studies are recommended.

## Conflicts of interest

None disclosed.
